# Effects of Bacterial Fermentation on the Biochemical Constituents and Antioxidant Potential of Fermented and Unfermented Soybeans Using Probiotic *Bacillus subtilis* (KCTC 13241)

**DOI:** 10.3390/molecules22122200

**Published:** 2017-12-11

**Authors:** Muhammad Waqas Ali, Il-Doo Kim, Saqib Bilal, Raheem Shahzad, Muhammad Tariq Saeed, Bishnu Adhikari, Rizwana Begum Syed Nabi, Jeong Rae Kyo, Dong-Hyun Shin

**Affiliations:** 1 School of Applied Biosciences, Kyungpook National University, Daegu 41566, Korea; waqasali3515@yahoo.com (M.W.A.); saqib043@yahoo.com (S.B.); raheemshehzad@ymail.com (R.S.); bishnu.adhk@gmail.com (B.A.); ruhii.syed@gmail.com (R.B.S.N.); rhkddms1128@naver.com (J.R.K.); 2 Institute of International Research & Development, Kyungpook National University, Daegu 41566, Korea; ildookim@hanmail.net; 3 Department of Agronomy, University of Agriculture Faisalabad, Faisalabad 38040, Pakistan; mtsuaf@gmail.com

**Keywords:** soybean, isoflavones, phenolic contents, antioxidant activity, *cheonggukjang*

## Abstract

Fermented soybeans, *cheonggukjang* (CKJ), are considered to be more wholesome than soybeans in Korea. To select the best soybean cultivar for making functional CKJ, a comparison was made between the biological activities of four soybean cultivars in their unfermented soybean (UFS) and CKJ states. Changes in 1,1-diphenyl-2-picrylhydrazyl (DPPH) radical-scavenging activity, 2,2′-azino-bis (3-ethylbenzothiazoline-6-sulphonic acid) (ABTS) assays, superoxide dismutase (SOD)-like activity, total phenolic compounds, total amino acids, and isoflavones were investigated. The levels of DPPH, ABTS, SOD-like activity, and total phenolic compounds increased in CKJ among all cultivars. The isoflavone aglycone and total amino acids showed the highest amount in CKJ prepared from soybean cultivar *Aga 3*. These results suggest that the improved antioxidant activity of CKJ in all cultivars might occur because of the higher levels of aglycones and total phenolic compounds achieved during fermentation. Moreover, CKJ prepared from soybean cultivar *Aga 3* showed higher antioxidant activity than the other cultivars and so can be considered for the commercial production of functional foods in the future.

## 1. Introduction

Reactive oxygen species (ROS) and oxygen free radicals, such as superoxide anion radicals, hydroxyl radicals, peroxides, and singlet oxygen, are produced in the human body when antioxidative defence systems become imbalanced. These radicals cause lipid peroxidation, which is the main reason for the deterioration of food. ROS are also a source of cell and tissue damage, such as cancer, cirrhosis, osteoporosis, diabetes, and ageing, through oxidising biological molecules [1]. Internally and externally produced antioxidants such as catalase, glutathione peroxidase, superoxide dismutase, ascorbic acid, and polyphenols work as defences against ROS [2]. Internally produced antioxidants alone are not adequate for a human body to prevent damage from ROS, therefore it is essential to get additional antioxidants from external sources like food.

Soybeans (*Glycine max* L.) have been a major food in Asian countries for thousands of years. They have high lipid and protein content and a balanced distribution of amino acids. They are also of vital importance due to containing physiologically active compounds that act as phytochemicals [3]. Soybeans are considered a rich source of many potent antioxidants, such as isoflavones, tocopherol, saponins, and phytic acids [4]. Soybeans contain different isoflavones and these isoflavones present in four different chemical forms: malonylglucosides (malonyldaidzin, malonylgenistin, and malonylglycitin), glucosides (daidzin, genistin, and glycitin), acetylglucosides (acetlyldaidzin, acetylgenistin, and acetylglycitin), and aglycones (daidzein, genistein, and glycitein); and have therapeutic roles against various lethal diseases such as cancer and cardiovascular disease caused by oxidative stress [5]. These functional compounds have high activity when soybeans are fermented using potential probiotics [6].

Microorganisms are seen as a source of antioxidant production in fermented foods, including tempeh produced by *Rhizopus oligosporus* in Indonesia [7]; Chinese *douche* produced by *Aspergillus oryzae*; Chinese *sufu*; Japanese *miso* produced by *Saccharomyces rouxii*; Japanese *natto*; and *kanjang*, *doenjang*, and *cheonggukjang* (CKJ) in Korea [8]. CKJ is a famous Korean fermented soybean that is consumed as a major food source [9]. Various studies have reported CKJ as being one of the best sources of protein, lipids, and hydrolyzed peptides, as well as being a health-promoting food [10]. It is also famous due to its fibrous texture, sticky and unique flavour, promising antioxidant, antigenotoxic, antimicrobial, anticancer, and antidiabetics activities, as well as its ability to control cholesterol levels in the body [11,12]. In addition, several studies have reported that the intake of fermented soybeans is related to a reduction in breast and prostate cancer rates in Asians [13].

On the basis of the aforementioned qualities of CKJ, this study aimed to identify and determine the best Korean soybean cultivar for producing *cheonggukjang* with superior phytochemical content and biological activity, using probiotic *Bacillus subtilis* (KCTC 13241) for the fermentation of the soybeans. The main focus of our research was CKJ prepared by the fermentation of soybeans at 42 °C with the microbial organism *B. subtilis*. For this purpose, four different soybean cultivars, *Aga 3*, *Saedanbeck*, *Pungsannamul*, and *Daewon*, were selected to evaluate the resulting CKJ for its phytochemical content, including isoflavones, total phenolic contents (TPC), and amino acid composition, as well as variation in the viability rates of cells in the CKJ. Additionally, the in vitro antioxidative activities of CKJ were investigated to assess their role in oxidative stress.

## 2. Results and Discussion

### 2.1. Viable Cell Number and pH of Unfermented Soybeans and Cheonggukjang

The viable cell number and pH of CKJ prepared with four different cultivars during fermentation by *B. subtilis* (KCTC 13241) increased (Table 1). The viable cell numbers in UFS made with *Daewon*, *Saedanbeck*, *Aga 3*, and *Pungsannamul* were 4.85, 4.81, 5.18, and 5.08 (log CFU/g), and increased following fermentation to 10.21, 10.11, 10.46, and 10.32, respectively. This change might be due to the fermentation period. In a previous study, it was reported that viable cell numbers markedly increased after fermentation [14], which is in strong agreement with our study. The pH of CKJ also increase into the range of 7.02–8.58 after 48 h fermentation. The increase in pH was a result of the breakdown of proteins into amino acids and the subsequent release of ammonia. These amino acids were utilised by the fermenting bacteria [14,15,16].

### 2.2. Antioxidant Activity of Unfermented Soybeans and Cheonggukjang

**Figure 1:**
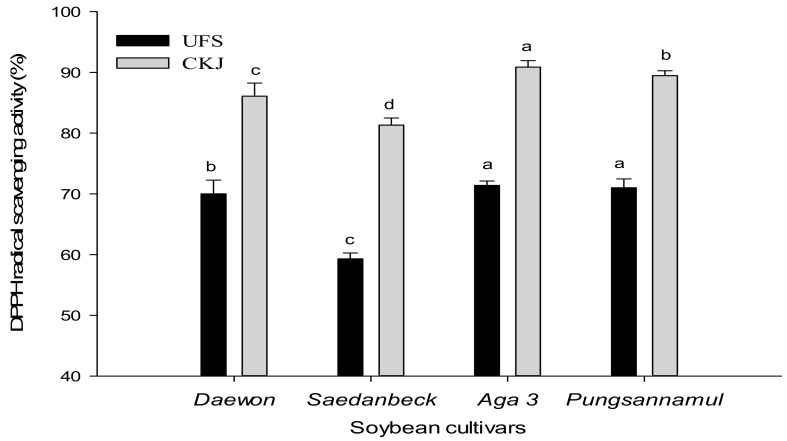
Changes in DPPH radical-scavenging activity between UFS and CKJ fermented by *Bacillus subtilis* (KCTC 13241) in different soybean cultivars. All values are means of three independent experiments. Means with different lowercase letters (a, b, c, and d) represent significant differences among cultivars by Duncan’s multiple range tests (DMRT) (*p* < 0.05).

The DPPH radical-scavenging assay, based on an electron-transfer reaction, is one of the most effective and widely-used assays for measuring the antioxidant activity of food and plant materials. In the presence of antioxidants, these free radicals form a colourless solution [17]. The DPPH scavenging capacity of samples is implied by the degree of reduction. The electron-donating activity of UFS and CKJ were tested in our study. Figure 1 represents the percentage of DPPH radical-scavenging activity in all samples. The DPPH percentage of UFS was in the range of 59.10–70.91% at 0 h fermentation, and increased to being in the range of 81.48–90.90% after 48 h fermentation. The antioxidant activity of CKJ was in the sequence *Aga 3* > *Pungsannamul* > *Daewon* > *Saedanbeck* according DPPH radical-scavenging assay results. After 48 h fermentation, these cultivars showed 19.54%, 18.38%, 16.11%, and 22.38% higher DPPH activity, respectively. The cultivar *Aga 3* exhibited 71.35% DPPH activity at 0 h fermentation, and after 48 h fermentation it rose to 90.90%. The next highest cultivar was *Pungsannamul*, which and showed 70.91% DPPH activity at 0 h fermentation and 89.29% at 48 h. The lowest DPPH percentage was measured in *Saedanbeck* with DPPH activity values of 59.10% and 81.48% for UFS and CKJ, respectively. These results exhibited higher antioxidant activities than those reported by *Cheongja* [18], and this may be due to different cultivars or microorganisms. Yee et al. [10] suggested that CKJ and its constituents show significant antioxidant activity. The rise in the radical-scavenging activity after fermentation depends on the isoflavone and total phenolic contents produced during fermentation by the potential probiotic *B. subtilis* CS90 [14].

**Figure 2:**
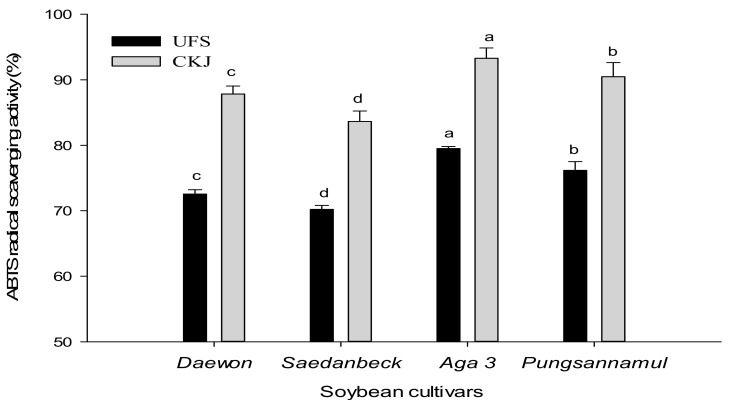
Changes in ABTS radical-scavenging activity between UFS and CKJ fermented by *Bacillus subtilis* (KCTC 13241) in different soybean cultivars. All values are means of three independent experiments. Means with different lowercase letters (a, b, c, and d) represent significant differences among cultivars by Duncan’s multiple range tests (DMRT) (*p* < 0.05).

ABTS radical-scavenging activity has been mostly used as an assessment of the antioxidant activity of food [19]. The antioxidant activity of extracts containing hydrophilic compounds is measured by ABTS assay. A stable ABTS radical, which has blue-green chromophore absorption, is produced by the oxidation of ABTS with potassium persulfate. The ABTS assay is based on the discoloration of the ABTS assay solution by measuring the reduction in absorbance at 734 nm [20]. The change in ABTS radical-scavenging activity for UFS and CKJ is shown in Figure 2. All cultivars made from CKJ had a higher percentage of ABTS radical-scavenging activity than UFS. These percentages ranged from 70.30 to 79.49% in UFS and 83.36 to 93.22% in CKJ, and cultivars were ranked: *Aga 3* > *Pungsannamul* > *Daewon* > *Saedanbeck*. The ABTS radical-scavenging activity in CKJ increased by 1.21-, 1.19-, 1.18-, and 1.19-fold in *Aga 3*, *Pungsannamul*, *Daewon*, and *Saedanbeck* respectively. However, like in the DPPH assay, CKJ prepared from *Aga 3* cultivar showed significantly (*p* < 0.05) greater ABTS radical-scavenging activity (93.22%) compared to those of the other cultivars. This change might be due to fermentation. Our results are similar to a previous study in which a *Tae-Kwang* soybean cultivar was reported and inoculated with *B. subtilis* W42 and *B. amyloliquefaciens* [21]. Additionally, another study reported that levels of ABTS radical activity in CKJ prepared from *Seoritae* and *Seormoktae* increased from 70.81% and 64.87% at 0 h fermentation, to 91.06% and 81.12% at 48 h fermentation, respectively [22]. These results are strongly supportive of our findings.

**Figure 3:**
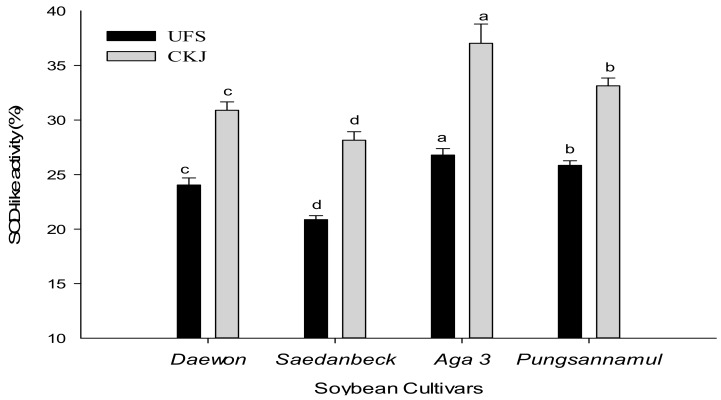
Changes in SOD-like activity between UFS and CKJ fermented by *Bacillus subtilis* (KCTC 13241) in different soybean cultivars. All values are means of three independent experiments. Means with different lowercase letters (a, b, c, and d) represent significant differences among cultivars by Duncan’s multiple range tests (DMRT) (*p* < 0.05).

Organic low molecular weight substances have SOD-like activity, including anti-aging properties and antioxidant effects. SOD-like activity is a natural antioxidant that removes active oxygen and maintains superoxide radicals [16]. The SOD-like activity of UFS was in the range of 20.87–26.76% and in CKJ its range was 28.20–36.90% (Figure 3). CKJ prepared from *Aga 3* exhibited the highest statistically significant (*p* < 0.05) SOD activity (36.90%) at 48 h fermentation and its activity in an UFS state was 26.76%, while CKJ of the *Saedanbeck* variety exhibited the lowest (28.20%) activity. Figure 3 shows that the SOD-like activities of UFS are increased in CKJ. A previous study reported that CKJ prepared from the *Taekwang* soybean cultivar showed 10% SOD-like activity at 1000 ppm in a methanol extract and 17.50% SOD-like activity at 1000 ppm in a water extract [23]. In our study, we found that the SOD activities of our cultivars were higher than in the aforementioned reports. The SOD-like activity of CKJ prepared from the *Cheongja* soybean cultivar was reported at around 31.54%, which is slightly higher than the results of our least-expressed cultivar, *Saedanbeck* [24]. We found that antioxidant activities, such as DPPH, ABTS, and SOD-like activity were also influenced by solvent extraction and bacterial strains for fermentation purposes. Additionally, the antioxidant activities might have been affected by the presence of either lower or higher levels of specific agents in the extracts such as isoflavone or phenolic contents.

### 2.3. Total Phenolic Contents of Unfermented Soybeans and Cheonggukjang

**Figure 4:**
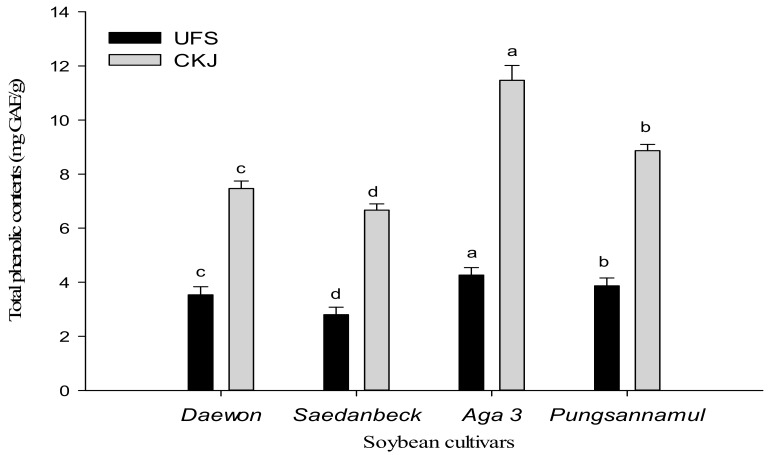
Changes in total phenolic contents (TPC) between UFS and CKJ fermented by *Bacillus subtilis* (KCTC 13241) in different soybean cultivars. All values are means of three independent experiments. Means with different lowercase letters (a, b, c, and d) represent significant differences among cultivars by Duncan’s multiple range tests (DMRT) (*p* < 0.05).

Phenolic compounds are secondary metabolites that are present in plants and have beneficial activities. They may scavenge free radicals based on their electron donor ability. They are important in the food industry due to their lipid peroxidation-reducing ability [25]. Total phenolic content was identified in the extracts of UFS and CKJ as shown in Figure 4. Significant (*p* < 0.05) differences were identified between different cultivars and between UFS and CKJ. Total phenolic content predominantly increased in CKJ during 48 h of fermentation when compared to UFS. Moreover, among the four cultivars, CKJ prepared from *Aga 3* showed the highest (11.51) value of total phenolic content. The total phenolic contents for both UFS and CKJ were in the following order: *Aga 3* > *Pungsannamul* > *Daewon* > *Saedanbeck* with values of 4.29, 3.78, 3.49, and 2.82 for UFS, and 11.51, 8.84, 7.49, and 6.71 for CKJ, respectively. These findings were comparable to previously-reported results on unfermented soybeans and *cheonggukjang*. A previous study reported that total phenolic content increased during soybean fermentation of *natto* into *cheonggukjang* [14]. Shin et al. [26] also reported that the total phenolic contents of *cheonggukjang* prepared with brown soybeans increased when the probiotic *Bacillus subtilis* used. This study indicated that *B. subtilis* (KCTC 13241) has strong potential for the biotransformation of soybean biopolymers into beneficial polyphenols during fermentation. CKJ prepared from *Aga 3* was detected to be prominent in having a high level of phenolic content. CKJ of *Aga 3* was also noted in exhibiting a high range of antioxidant activities. The results indicate that the presence of high levels of isoflavones might responsible for its influential role as an antioxidant.

### 2.4. Isoflavone Composition of Unfermented Soybeans and Cheonggukjang

Isoflavones are flavonoids present in soybean cultivars and are classified as phytoestrogens since their structures resemble that of estrogen and they have a weak affinity for the estrogen receptor [27]. In the current study, we analysed the effects of the bacterial strain *B. subtilis* (KCTC 13241) on the isoflavone composition of soybean cultivars. The isoflavone composition of four different soybean cultivars in UFS and CKJ states is listed in Table 2. Glycoside isoflavones, including daidzin, genistin, and glycitin, decreased in concentration in CKJ when compared to UFS, and aglycone isoflavones such as daidzein, glycitein, and genistein, significantly (*p* < 0.05) increased in CKJ. The concentration of these isoflavones was also different among different cultivars. *Aga 3* showed a remarkable difference compared to cultivars; it had 1078.50 µg/g total isoflavone concentration in its UFS state and this decreased in its CKJ state to 785.99 µg/g. *Saedanbeck* had the lowest concentration of total isoflavones among all cultivars.

The composition of these isoflavones depends upon the processing techniques used during fermentation, the microorganisms used, and the soybean cultivars used. Previous studies showed that the values of isoflavones in soybean foods, such as *douche*, *cheonggukjang*, and *tofu*, become low depending on the processing technique useds [14,28]. Cho et al. [14], reported that the total isoflavone composition in CKJ decreased by around 64%, from 2923.21 µg/g to 1051.59 µg/g, after 60 h of fermentation. In another study, the total isoflavone content decreased from 1055 µg/g in UFS (0 h fermentation) to 870 µg/g (36 h fermentation) during fermentation by *B. subtilis* [29]. In our study, the total isoflavone content decreased by about 222.59, 160.90, 161.72, and 292.51 μg/g, after 48 h of fermentation of *Daewon*, *Saedanbeck*, *Aga 3*, and *Pungsannamul*, respectively.

Generally, isoflavones in soybean cultivars are present in the form of glycoside and are changed into aglycones during fermentation by microorganism activity [14,30]. Another study showed that aglycone isoflavones, such as daidzein, glycitein, and genistein, increased and glycoside isoflavones decreased in *cheonggukjang* as fermentation time increased [22]. It stated that the total isoflavones in food made under normal cooking conditions was not reduced, whereas food made under high-temperature conditions resulted in an increase the level of aglycones and a decrease in total isoflavone content [26]. In our study, we prepared UFS and CKJ by cooking the soybeans at 121 °C for 30 min followed by 0 h and 48 h fermentation respectively. We found that *B. subtilis* (KCTC 13241) fermentation had the effect of increasing the aglycone content and decreasing the glycoside content. These results also demonstrated that isoflavone composition and concentration in soybean foods varies depending on the soybean variety, addition of microorganisms, cooking conditions, and fermentation duration.

### 2.5. Free Amino Acid Composition

Twenty amino acids, including aspartic acid (Asp), threonine (Thr), serine (Ser), glutamic acid (Glu), α-amino adipic acid (Aaa), glycine (Gly), alanine (Ala), citrulline (Cit), α-amino-*n*-butyric acid (Aba), valine (Val), methionine (Met), cystathionine (Cys), isoleucine (Iso), leucine (Leu), tyrosine (Tyr), phenylalanine (Phe), β-alanine (Bal), β-amino isobutyric acid (Bam), γ-amino-*n*-butyric acid (Gaba), and ethanol amine (Eth), were analyzed in UFS and CKJ as shown in Table 3. Most of the amino acids, such as Asp, Thr, Glu, Gly, Ala, Val, Met, Cys, Iso, Leu, Tyr, Phe, Bal, Bam, and Eth, were identified at higher levels in CKJ among all cultivars than in UFS, while Cit showed higher values in UFS. The value of Ser in CKJ decreased in *Aga 3* and *Pungsannamul* and increased in *Daewon* and *Saedanbeck*.

Gaba, naturally present in soybeans, was detected in all cultivars. It showed higher values in CKJ than in UFS among all cultivars, and *Daewon* had the highest value in CKJ. Gaba is produced in soybean foods by the decarboxylation of l-Glu, which is catalysed by glutamic decarboxylase [31]. Gaba has physiological roles, such as regulating cardiovascular functions and controlling blood pressure. However, Ser, Aaa, and Aba levels were low in all cultivars in both CKJ and UFS. Thr boosts the immune system and promotes the growth of thymus glands [32]. Leu helps in regulating oxidative glucose utilisation by skeletal muscles and also facilitates bone and skin wound healing by modulating enkephalins [33,34].

Asp, Glu, and Ala play important roles in human health: Ala maintains glucose and nitrogen balance through the glucose–alanine cycle in the human body, Asp is beneficial in higher plants and acts as a precursor for the synthesis of several amino acids, and Glu is used for the treatment of various neurological disorders and is also famous for boosting the taste of food [35,36].

Overall, the value of amino acids in CKJ was higher than in UFS and a significant increase in amino acid levels was seen in CKJ after 48 h fermentation. Choi et al. [24] reported that the concentration of amino acids was higher in initial fermentation in *cheonggukjang* prepared from germinated soybeans. A previous study also showed that protein content decreased over the fermentation time 24–48 h in *cheonggukjang* made using *Bacillus licheniformis*. Amino acids such as Gly, Lys, and Glu can positively affect the quality of *cheonggukjang*, and Tyr and Phe are responsible for the savoury taste of *cheonggukjang* [37]. *Aga 3* and *Pungsannamul* were identified as the best cultivars when we considered total free amino acid levels and these cultivars can be recommended for use in the production of the best CKJ.

## 3. Materials and Methods

### 3.1. Chemical

Glacial acetic acid, Folin-Ciocalteu phenol reagent, 2,2-azinobis (3-ethyl-benzothiazoline-6-sulfonic acid) diammonium salt (ABTS), potassium persulfate, ferric chloride, sodium acetate, 2,4,6-tripyridyl-s-triazine (TPTZ), rutin, isoflavone aglycones, including daidzein, genistein, and glycitein, were obtained from Sigma-Aldrich Chemical Co. (St. Louis, MO, USA), and three isoflavone glycosides, including genistin, daidzin, and glycitin, were purchased from Indofine (Hillsborough, FL, USA). HPLC-grade H_2_O, methanol, and acetonitrile were purchased from Fisher Scientific (Fairlawn, OH, USA). All chemicals were of analytical grade.

### 3.2. Soybean Cultivars and Microorganisms

Four soybeans cultivars (*Daewon*, *Saedanbeck*, *Aga 3*, and *Pungsannamul*) were collected from the Genetics and Plant Breeding Lab, School of Applied Biosciences. A previously-isolated strain of *Bacillus subtilis* (KCTC 13241) from traditionally-fermented *cheonggukjang* [38] was used for the preparation of CKJ. The *Bacillus subtilis* was maintained in the laboratory on nutrient agar slants. For inoculum preparation, the activated culture was streaked onto a nutrient agar slant and incubated at 37 °C for 16 h. The cells were harvested in sterile distilled water, and after adjusting to a concentration of 10^7^–10^8^ total cells mL^−1^, the suspension was used to inoculate cooked soybeans for fermentation.

### 3.3. Preparation of Cheonggukjang

500 g of soybeans of each cultivar were sorted, washed, soaked in water for 12 h at 25 °C, drained, and steam-heated for 30 min at 121 °C. The cooked mass was cooled to approximately 40 °C, inoculated with a 3% (*v*/*w*) *B. subtilis* culture medium (10^7^–10^8^ CFU/mL), and fermented in an incubator for 48 h at 42 °C [39].

### 3.4. Viable Cell Number and pH

A 1 g of sample was mixed with 9 mL 0.85% NaCl solution, and diluted suspensions (0.1 mL portions) were spread on a tryptic soy agar (TSA) plate. The plates were incubated at 37 °C for 24 h, after which colony counts were carried out. A 10 g portion of the CKJ samples, with different cultivars, was dissolved in 90 mL of distilled water at room temperature for 1 h and was then filtered through Whatman No. 4 filter paper (Millipore, Billerica, MA, USA). The filtrate pH was measured by a pH meter (PHS-3BW, Bante, Shanghai, China) [14].

### 3.5. Sample Extraction

Extractions were carried out by Xu and Chang [40] with some modifications. Fermented soybeans (CKJ) underwent freeze-drying and then were finely ground by a grinding machine (Speed Rotor Mill, Model KT-02A, Seishin, Fukuoka, Japan). Unfermented soybeans (UFS) were directly ground without freeze-drying. The ground flour was used for extraction. A 1 g of sample with 10 mL of 80% methanol extraction solvent (triplicate) was incubated at 25 °C for 24 h. The incubated extracts were centrifuged at 3000 rpm for 15 min. The supernatants were filtered through a 0.45 mm Minipore PVDF filter (Schleicher & Schuell, GmbH, Dassel, Germany). The filtrate was used for assays of total phenolic content (TPC) and antioxidant activity.

### 3.6. DPPH Radical-Scavenging Activity

The free radical-scavenging activity of UFS and CKJ was measured following the protocol described by Blois [41] with some modifications. Freshly prepared DPPH solution in methanol (99.90%) was used for the experiment. A mixture of equal volumes of methanol-extracted samples and freshly prepared 0.1% DPPH solution was left in dark for 30 min. The absorbance was measured at 517 nm using a Multiskan GO Microplate Spectrophotometer (Thermo Fischer Scientific, Vantaa, Finland). An equal proportion of DPPH and methanol was mixed to measure the absorbance of the control. The DPPH radical-scavenging activity was calculated from the absorbance according to the following equation:

DPPH radical-scavenging activity (%) = [1 − ((S − S_0_)/C)] × 100 (1)

where S = absorbance of DPPH and sample, S_0_ = absorbance of methanol and sample, C = absorbance of control.

### 3.7. ABTS Radical-Scavenging Activity

A spectrophotometric assay of ABTS radical-scavenging activity was performed as described in our previous study [42]. Briefly, ABTS cation radicals were generated by reacting 7 mM ABTS solution with 140 mM potassium persulfate, which was allowed to stand in the dark at room temperature for 16 h. Before usage, the solution was diluted to achieve an absorbance of 0.7 ± 0.02 at 734 nm with 50% ethanol. To determine the scavenging activity, the ABTS reagent was mixed with the sample and the absorbance at 734 nm was measured 3 min after the initial mixing, using 50% ethanol as the blank. Ascorbic acid was used as a positive control. The scavenging capability of the ABTS radical was calculated using the following equation:

Inhibition rate (%) = (Control O.D/Sample O.D) × 100(2)

### 3.8. Superoxide Dismutase (SOD)-Like Activity

Assessment of SOD-like activity was performed using the method described by Cho et al. [43]. Tris-HCl buffer (50 mM, 0.3 mL; hydroxymethyl amino-methane, 10 mM EDTA, pH 8.5) and 0.2 mL of 7.2 mM pyrogallol were applied to a 0.2 mL extracted sample and reacted at 25 °C for 10 min. The reaction was stopped by applying 1 mL of 1 N HCl. The amount of pyrogallol oxidised during the reaction was measured at an absorbance at 420 nm. The difference in the absorbance between the experimental group and the control group was recorded as a percentage.

SOD-like activity (%) = [(Control − Sample)/Control] × 100(3)

### 3.9. Isoflavone Analysis

Isoflavone analysis was performed using the method described by Kuan [44], with slight modifications. Briefly, 1 g of each sample was defatted with 5 mL hexane and then extracted for 1 h at room temperature with 5 mL acetonitrile, 4.5 mL water, and 0.5 mL of the internal standard THB (0.5 mg/mL). After centrifugation at 10,000× *g* for 20 min, the supernatant was collected through filter paper No 42. The extraction solvent was evaporated at 35 °C to dry. Ten µL of filtrate was injected into an HPLC system equipped with a Symmetry C18 column after the system had been equilibrated at 30 °C. The UV detector was stabilized with a mobile phase (A: Acetonitrile, B: HPLC water (1% acetic acid), A: 5% (1 min), 5 → 35% (50 min), 35 → 5% (5 min), 5% (15 min) at a flow rate of 1.0 mL/min). The effluent was detected at 254 nm. The isoflavones were identified by their retention times of standard addition, and their contents were calculated by comparing their peak areas with those of standards.

### 3.10. Total Phenolic Contents (TPC)

Total phenolic contents were measured by the Folin-Ciocalteu method [45]. Powdered sample (1 g) was extracted with 10 mL methanol in a shaking incubator at 25 °C for 24 h. The mixture was centrifuged at 3000 rpm for 15 min and the supernatant liquid was filtered through a 0.2 µm syringe filter (Water, Milford, MA, USA). Fifty microliters of methanolic extract was added to 1 mL aqueous solution of 2% sodium carbonate and was left for 3 min. The mixture was mixed with 50 µL of 1 N Folin–Ciocalteu reagent and left in the dark for 30 min at room temperature. The absorbance was read at 750 nm using a Multiskan GO Microplate Spectrophotometer (Thermal Fischer Scientific, Vantaa, Finland). The standard calibration curve was plotted using gallic acid. The polyphenol content was expressed as milligram gallic acid equivalent per gram of sample (mg GAE/g).

### 3.11. Free Amino Acid Composition

The free amino acid composition of UFS and CKJ was analysed as described by Waqas [46]. Briefly, approximately 1 g of ground sample from each was hydrolyzed in 5 mL of 6 N HCl under a vacuum in an ampulla tube for 24 h at 110 °C. The suspension was then filtered and evaporated under a vacuum. The solid residue was dissolved in 2 mL of deionized water and evaporated twice. The final residue was dissolved in 10 mL of 0.01 N HCl and filtered through a 0.45 µm filter membrane using an automatic amino acid analyser (L-8900 Hitachi, Tokyo, Japan). An amino acid standard mixture solution (type H) for automatic amino acid analysis was purchased from Wako Pure Chemical Industries, Ltd. (Osaka, Japan) and used for the accurate analysis of amino acid composition. All of the samples were run in triplicate and expressed in micrograms per gram (µg/g).

### 3.12. Statistical Analysis

Data were subjected to analysis of variance by using SAS version 9.3 (SAS Institute Inc., Cary, NC, USA). The separation of treatment means was conducted using Duncan’s multiple range tests (DMRT) at the 95% confidence level. The results were expressed as the means ± SD (standard deviation) of three replicates. SigmaPlot was used for graphical presentations.

## 4. Conclusions

Antioxidant activities such as DPPH, ABTS, and SOD-like activity, were markedly increased among all soybean cultivars during 48 h of fermentation. The isoflavone aglycones were significantly increased while glycosides decreased during fermentation. These changes have been documented for the first time for the microorganism *Bacillus subtilis* (KCTC 13241). These developments might be due to the β-glucosidase produced by *B. subtilis*. This research also suggested that the high antioxidant activity of CKJ might be related to the higher isoflavone aglycone levels and higher total phenolic contents that increase during fermentation. Among all the studied soybean cultivars, CKJ prepared from *Aga 3* proved to be the most markedly significant in antioxidant activity, total phenolic content, and isoflavones levels, as well as free amino acid composition. Additionally, soybean cultivar *Aga 3* could be recommended for the commercial production of fermented soybean. Furthermore, all other cultivars showed notable nutritional and biological contents, such as isoflavones, phenolic contents, antioxidants, and free amino acids. The difference between UFS and CKJ might be due to the fermentation period (48 h) and differences among cultivars might be due to genotype, crop location, crop year, storage period, climatic conditions, seed size, and colour. Moreover, further research is essential for examining the effects of isoflavones, determination of the molecular mechanisms responsible for the antioxidant activitie, and amino acid profile modification for health benefits and pharmaceutical drugs.

## Figures and Tables

**Table 1:** Viable cell number and pH of unfermented soybeans and *cheonggukjang* fermented by *Bacillus subtilis* (KCTC 13241).

Cultivars	Samples ^1^	Viable Cell Numbers (log CFU/g)	pH
*Daewon*	UFS ^2^	4.85 ± 0.72 ^a^	5.87 ± 0.18 ^b^
	CKJ ^3^	10.21 ± 0.84 ^a^	7.79 ± 0.55 ^b^
*Saedanbeck*	UFS	4.81 ± 0.41 ^a^	5.19 ± 0.35 ^c^
	CKJ	10.11 ± 1.03 ^a^	7.02 ± 0.22 ^c^
*Aga 3*	UFS	5.18 ± 0.80 ^a^	6.26 ± 0.20 ^a^
	CKJ	10.46 ± 1.21 ^a^	8.58 ± 0.28 ^a^
*Pungsannamul*	UFS	5.08 ± 0.37 ^a^	6.19 ± 0.14 ^a^
	CKJ	10.32 ± 0.36 ^a^	8.33 ± 0.12 ^a^

^1^ Values indicate the means of three replications (*n* = 3). Means with different lowercase letters (^a^, ^b^, and ^c^) indicate significant differences between unfermented soybeans and *cheonggukjang* by Duncan’s multiple range tests (DMRT) at the 95% confidence level. ^2^ UFS: unfermented soybeans. ^3^ CKJ: *cheonggukjang* fermented at 42 °C for 48 h.

**Table 2:** Changes in isoflavone contents between unfermented soybeans and *cheonggukjang* fermented by *Bacillus subtilis* (KCTC 13241) in different soybean cultivars.

Isoflavone µg/g	Sample ^1^	Soybean Cultivars			
		*Daewon*	*Saedanbeck*	*Aga 3*	*Pungsannamul*
Daidzin	UFS ^2^	145.60 ± 6.20 ^c^	126.54 ± 4.11 ^d^	347.04 ± 2.41 ^a^	236.39 ± 2.50 ^b^
	CKJ ^3^	47.70 ± 5.36 ^c^	26.84 ± 1.91 ^d^	162.05 ± 1.12 ^a^	130.68 ± 4.37 ^b^
Genistin	UFS	163.01 ± 3.19 ^c^	145.01 ± 2.76 ^d^	424.25 ± 4.35 ^a^	262.71 ± 4.12 ^b^
	CKJ	55.12 ± 4.11 ^c^	43.34 ± 4.03 ^d^	244.73 ± 2.59 ^a^	155.66 ± 3.46 ^b^
Glycitin	UFS	17.39 ± 2.24 ^c^	10.91 ± 1.20 ^d^	93.85 ± 2.00 ^a^	55.79 ± 2.22 ^b^
	CKJ	3.18 ± 0.90 ^c^	1.96 ± 0.39 ^d^	30.42 ± 2.50 ^a^	15.87 ± 1.97 ^b^
Daidzein	UFS	66.48 ± 2.59 ^c^	34.82 ± 3.43 ^d^	119.45 ± 3.75 ^a^	88.60 ± 0.70 ^b^
	CKJ	85.50 ± 1.42 ^c^	65.81 ± 2.58 ^d^	213.91 ± 4.17 ^a^	101.87 ± 2.36 ^b^
Glycitein	UFS	40.35 ± 1.14 ^c^	30.34 ± 2.97 ^d^	91.48 ± 1.94 ^a^	74.68 ± 2.39 ^b^
	CKJ	75.26 ± 3.74 ^c^	46.04 ± 2.49 ^d^	110.38 ± 2.45 ^a^	88.04 ± 3.87 ^b^
Genistein	UFS	3.18 ± 0.31 ^a^	1.32 ± 0.48 ^b^	2.43 ± 0.45 ^a,b^	3.17 ± 0.89 ^a^
	CKJ	7.64 ± 0.05 ^b^	4.16 ± 0.69 ^c^	24.50 ± 1.53 ^a^	8.63 ± 0.70 ^b^
Total isoflavones	UFS	436.00 ± 0.54 ^c^	348.94 ± 0.36 ^d^	1078.50 ± 1.12 ^a^	721.34 ± 0.65 ^b^
	CKJ	274.40 ± 0.23 ^c^	188.15 ± 0.43 ^d^	785.54 ± 0.87 ^a^	500.76 ± 0.87 ^b^

^1^ Values indicate the means of three replications (*n* = 3). Means with different lowercase letters (^a^, ^b^, ^c^, and ^d^) indicate significant differences between the unfermented soybeans and *cheonggukjang* by Duncan’s multiple range tests (DMRT) at the 95% confidence level. ^2^ UFS, unfermented soybean. ^3^ CKJ, *cheonggukjang* fermented at 42 °C for 48 h.

**Table 3:** Analysis of free amino acid composition in unfermented soybean and *cheonggukjang* fermented by *Bacillus subtilis* (KCTC 13241) in different soybean cultivars.

Amino Acids µg/g	Sample ^1^	*Daewon*	*Saedanbeck*	*Aga 3*	*Pungsannamul*
aspartic acid	UFS ^2^	99.38 ± 2.20 ^b^	43.39 ± 1.14 ^d^	138.33 ± 1.96 ^a^	56.52 ± 0.22 ^c^
	CKJ ^3^	443.95 ± 2.15 ^a^	368.56 ± 1.42 ^b^	288.69 ± 1.50 ^d^	320.98 ± 1.65 ^c^
threonine	UFS	47.95 ± 2.68 ^b^	5.20 ± 0.36 ^d^	79.22 ± 1.33 ^a^	39.38 ± 0.57 ^c^
	CKJ	171.72 ± 2.09 ^a^	139.46 ± 1.65 ^c^	143.79 ± 2.12 ^b^	111.39 ± 1.55 ^d^
serine	UFS	26.69 ± 1.30 ^b^	1.13 ± 0.83 ^d^	52.40 ± 1.28 ^a^	15.98 ± 3.42 ^c^
	CKJ	44.99 ± 1.29 ^a^	13.90 ± 1.47 ^b^	13.32 ± 0.53 ^b^	6.73 ± 0.60 ^c^
glutamic acid	UFS	531.55 ± 3.64 ^b^	381.82 ± 2.97 ^c^	603.74 ± 1.27 ^a^	348.73 ± 1.17 ^d^
	CKJ	1028.83 ± 2.19 ^a^	975.96 ± 0.78 ^b^	619.80 ± 2.31 ^c^	430.90 ± 0.84 ^d^
α-amino adipic acid	UFS	96.99 ± 4.46 ^b^	267.05 ± 0.53 ^a^	54.41 ± 0.97 ^c^	55.32 ± 1.58 ^c^
	CKJ	106.79 ± 0.76 ^b^	115.53 ± 1.56 ^a^	39.16 ± 2.41 ^d^	92.93 ± 3.26 ^c^
glycine	UFS	176.04 ± 1.03 ^b^	41.48 ± 1.00 ^d^	193.33 ± 1.69 ^a^	116.88 ± 3.03 ^c^
	CKJ	291.14 ± 2.20 ^a^	218.58 ± 1.04 ^b^	214.41 ± 0.52 ^c^	204.40 ± 2.71 ^d^
alanine	UFS	580.17 ± 1.45 ^a^	73.53 ± 0.50 ^d^	420.89 ± 2.99 ^b^	234.57 ± 1.00 ^c^
	CKJ	1017.40 ± 2.25 ^a^	544.99 ± 1.23 ^b^	542.55 ± 1.02 ^c^	392.15 ± 1.40 ^d^
citrulline	UFS	221.09 ± 3.43 ^c^	760.79 ± 1.53 ^a^	365.01 ± 0.64 ^b^	219.57 ± 2.79 ^d^
	CKJ	ND ^c^	291.41 ± 0.69 ^a^	ND ^c^	158.76 ± 1.83 ^b^
α-amino-*n*-butyric acid	UFS	20.87 ± 1.24 ^a^	ND ^c^	18.84 ± 1.27 ^b^	ND ^c^
	CKJ	12.50 ± 0.55 ^a^	10.98 ± 2.20 ^a,b^	9.92 ± 0.01 ^b^	10.55 ± 1.06 ^a,b^
valine	UFS	39.09 ± 3.51 ^c^	39.58 ± 1.01 ^c^	85.02 ± 1.34 ^a^	46.17 ± 0.47 ^b^
	CKJ	133.26 ± 1.18 ^b^	103.16 ± 0.82 ^c^	148.05 ± 1.19 ^a^	104.93 ± 1.17 ^c^
methionine	UFS	28.18 ± 3.54 ^a^	ND ^c^	28.95 ± 2.58 ^a^	24.45 ± 0.51 ^b^
	CKJ	102.36 ± 2.52 ^a^	76.07 ± 1.87 ^b^	77.29 ± 0.83 ^b^	68.69 ± 1.26 ^c^
cystathionine	UFS	15.95 ± 1.56 ^a^	ND ^c^	11.65 ± 1.99 ^b^	14.71 ± 2.26 ^a,b^
	CKJ	16.83 ± 1.86 ^a^	14.16 ± 2.55 ^a,b^	12.14 ± 0.82 ^b^	15.02 ± 1.46 ^a,b^
isoleucine	UFS	17.37 ± 1.93 ^d^	30.35 ± 0.72 ^b^	42.78 ± 0.59 ^a^	26.82 ± 2.51 ^c^
	CKJ	99.57 ± 1.59 ^b^	75.96 ± 1.67 ^c^	104.57 ± 2.91 ^a^	73.67 ± 0.37 ^d^
leucine	UFS	23.71 ± 3.67 ^c^	4.33 ± 0.80 ^d^	82.42 ± 2.57 ^a^	39.60 ± 0.50 ^b^
	CKJ	195.29 ± 2.46 ^b^	142.13 ± 1.87 ^d^	225.25 ± 0.75 ^a^	152.81 ± 1.59 ^c^
tyrosine	UFS	88.21 ± 2.47 ^b^	56.87 ± 0.66 ^d^	136.28 ± 1.45 ^a^	76.58 ± 1.22 ^c^
	CKJ	170.89 ± 3.89 ^c^	172.50 ± 1.87 ^b^	204.54 ± 1.00 ^a^	204.18 ± 0.33 ^a^
phenylalanine	UFS	43.11 ± 1.38 ^d^	47.47 ± 1.14 ^c^	119.95 ± 2.27 ^a^	80.77 ± 0.97 ^b^
	CKJ	184.06 ± 1.73 ^d^	189.72 ± 0.86 ^c^	224.30 ± 1.12 ^b^	235.76 ± 1.58 ^a^
β-alanine	UFS	126.44 ± 2.22 ^a^	35.32 ± 0.57 ^c^	43.78 ± 1.05 ^b^	43.44 ± 1.85 ^b^
	CKJ	161.16 ± 1.00 ^a^	105.92 ± 2.47 ^b^	60.75 ± 1.81 ^d^	101.36 ± 2.13 ^c^
β-amino isobutyric acid	UFS	30.80 ± 2.03 ^c^	46.71 ± 1.89 ^a^	21.88 ± 1.73 ^d^	39.06 ± 0.73 ^b^
	CKJ	56.61 ± 1.33 ^c^	71.27 ± 2.02 ^b^	41.55 ± 0.60 ^d^	78.31 ± 0.72 ^a^
γ-amino-*n*-butyric acid	UFS	663.83 ± 3.58 ^a^	107.43 ± 1.07 ^d^	515.72 ± 1.51 ^b^	316.93 ± 1.37 ^c^
	CKJ	853.14 ± 2.90 ^a^	724.61 ± 2.61 ^b^	611.31 ± 3.16 ^d^	617.14 ± 0.45 ^c^
ethanol amine	UFS	68.16 ± 0.87 ^a^	36.23 ± 1.07 ^c^	62.03 ± 1.41 ^b^	29.72 ± 1.34 ^d^
	CKJ	70.16 ± 1.29 ^a,b^	68.33 ± 1.24 ^b^	71.37 ± 0.99 ^a^	52.94 ± 2.29 ^c^
Total free amino acids	UFS	2595.34 ± 0.82 ^c^	2243.26 ± 1.24 ^d^	3371.00 ± 2.06 ^a^	3082.94 ± 2.43 ^b^
	CKJ	5879.04 ± 1.27 ^c^	5177.41 ± 1.23 ^d^	6989.48 ± 1.98 ^a^	6452.05± 2.81 ^b^

^1^ All values are means of determinations in three independent experiments. Means with different lowercase letters (^a^, ^b^, ^c^, and ^d^) indicate significant differences of unfermented soybean and *cheonggukjang* by Duncan’s multiple range tests (DMRT) at the 95% confidence level. ^2^ UFS, unfermented soybean. ^3^ CKJ, *cheonggukjang*.
